# Metagenomic data of bacterial communities associated with *Acropora* species from Phu Quoc Islands, Vietnam

**DOI:** 10.1016/j.dib.2023.108977

**Published:** 2023-02-14

**Authors:** Duong Huy Nguyen, Nhat Huy Chu, Yvan Bettarel, Jean-Christophe Auguet, Thierry Bouvier, Ha Hoang Chu, Van Ngoc Bui

**Affiliations:** a Institute of Biotechnology (IBT), Vietnam Academy of Science and Technology (VAST), Hanoi, Vietnam; b Graduate University of Science and Technology (GUST), VAST, Hanoi, Vietnam; c UMR MARBEC IRD-CNRS-IFREMER-Université Montpellier, Montpellier, France

**Keywords:** Bacterial diversity, Core microbiome, Coral reefs, Grazed *Acropora*, Illumina sequencing

## Abstract

*Acropora* is one of the most common coral genera found in Phu Quoc Islands, Vietnam. However, the presence of marine snails, such as the coralllivorous gastropod *Drupella rugosa,* was a potential threat to the survival of many scleractinian species, leading to changes in the health status and bacterial diversity of coral reefs in Phu Quoc Islands. Here, we describe the composition of bacterial communities associated with two species of *Acropora* (*Acropora formosa* and *Acropora millepora*) using the Illumina sequencing technology. This dataset includes 5 coral samples of each status (grazed or healthy), which were collected in Phu Quoc Islands (9°55′20.6″N 104°01′16.4″E) in May 2020. A total of 19 phyla, 34 classes, 98 orders, 216 families and 364 bacterial genera were detected from 10 coral samples. Overall, *Proteobacteria* and *Firmicutes* were the two most common bacterial phyla in all samples. Significant differences in the relative abundances of the genera *Fusibacter, Halarcobacter, Malaciobacter,* and *Thalassotalea* between grazed and healthy status were observed. However, there was no differences in alpha diversity indices between the two status. Furthermore, the dataset analysis also indicated that *Vibrio* and *Fusibacter* were core genera in the grazed samples, whereas *Pseudomonas* was the core genus in the healthy samples.

**Specifications Table**

**Table:** 

Subject	Microbiology and Metagenomics
Specific subject area	Coral microbiome
Type of data	Table, figure, and FASTQ files
How data were acquired	Amplicon metasequencing with of V3-V4 regions of the 16S rRNA genes using Illumina HiSeq platform, OTU clustering analysis was conducted using pipeline (version 1.8) – R Studio (version 4.2.1) and SILVA database (version 138.1)
Data format	Raw and analysed data
Parameters for data collection	10 mucus coral samples including 5 grazed samples and 5 healthy samples, were collected from species *Acropora formosa* and *Acropora millepora* in Phu Quoc Islands (9°55′20.6"N 104°01′16.4"E), Vietnam in May 2020. For comparative analysis, samples were divided into two groups (grazed and healthy) according to the coral's health status.
Data source location	Institution: Institute of Biotechnology, Vietnam Academy of Science and Technology City: Hanoi Country: Vietnam
Data accessibility	In a public repository: NCBI and Mendeley Data The raw data are available in NCBI Repository name: SRA Data identification number: BioProject No. PRJNA890553; BioSample: SAMN31280571 and SAMN31280572; Run: SRR21916498, SRR21916499, SRR21916500, SRR21916524, SRR21916525, SRR21916496, SRR21916497, SRR21916501, SRR21916502, SRR21916513 Mendeley data: https://doi.org/10.17632/6kmck96frj.1

## Value of the Data

• This dataset provides the description of bacterial diversity and community composition associated with *Acropora formosa* and *Acropora millepora* corals from Phu Quoc Island, Vietnam.
• The dataset is a valuable source for comparison of bacterial communities between various coral species, as well as the host's status.
• Based on understanding of these bacterial communities, the data might be utilized for metabolic and functional prediction of microbial communities in corals, specially in the stress conditions caused by coral predators.

## 1 Objective

This dataset is an important part of the project, namely **Ecogenomics of viruses in two coral reefs in Vietnam: Phu Quoc and Con Dao Islands.** In this project, we collected some microorganims for investigation of the microbial diversity and ecology associated with coral reefs in Phu Quoc and Con Dao Islands. These data included viruses, bacteria, archaea, and microeukaryota, which were collected from healthy, bleached, and grazed coral species. In this article, we provide the bacterial dataset for comparison and evaluation of bacterial communities associated with two genera, *Acropora formosa* and *Acropora millepora.* This provides another aspect of bacterial diversity, which is affected not only by abiotic factors but also by biotic factors such as coral predators.

## 2 Data Description

The composition of bacterial communities living in *Acropora formosa* and *Acropora millepora* was investigated based on 16S rRNA gene sequencing. After removing chimeric, singletons, mitochondrial and chloroplast sequences, a total of 218,703 reads, with a median of 27,356 and a mean of 21,870 sequences per sample, were obtained from 10 coral mucus samples.

**Fig. 1:**
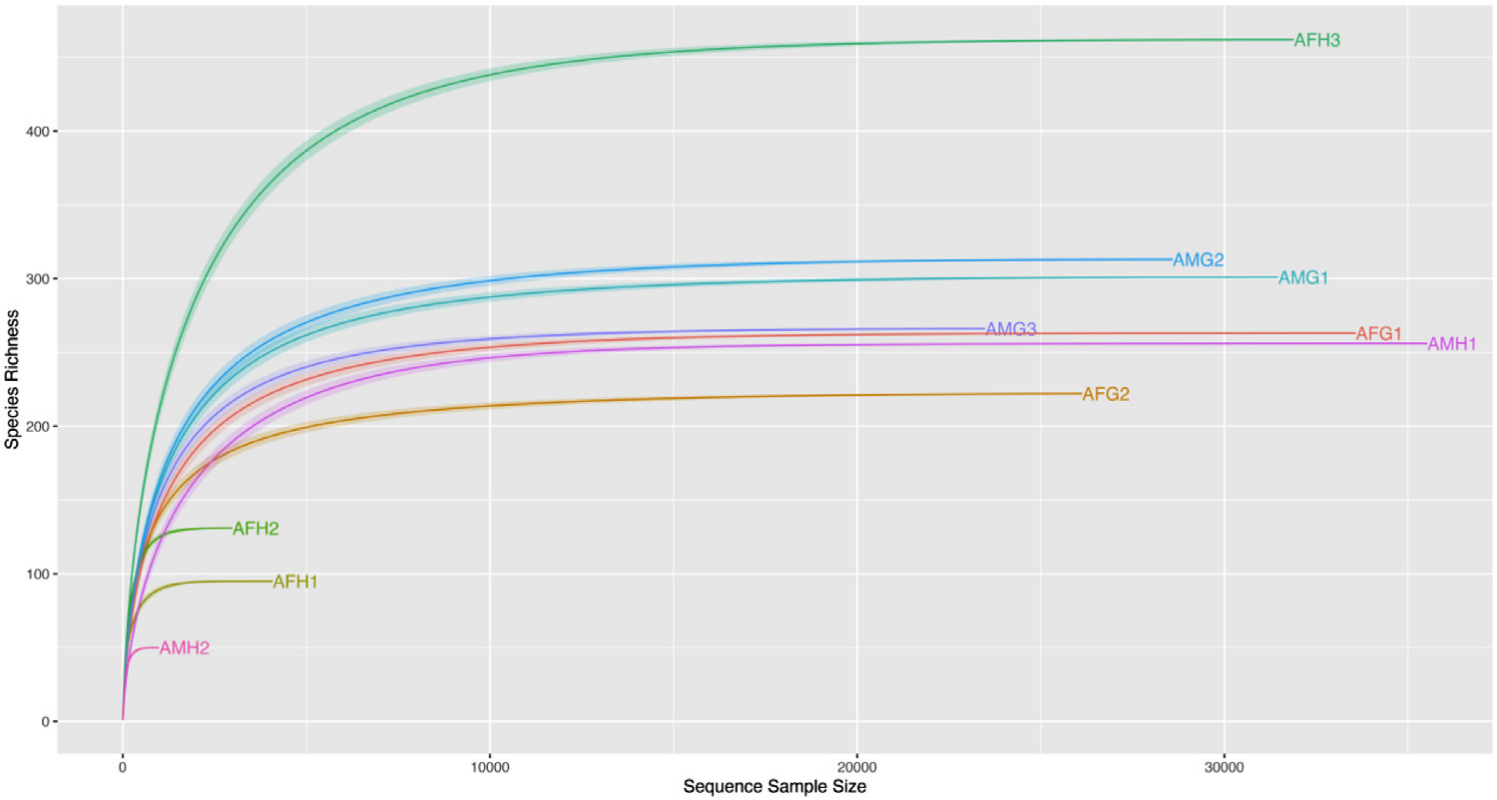
Rarefaction curves for metagenomic datasets of 10 coral samples from Phu Quoc Islands.

The species richness varied considerably across the samples. However, the rarefaction curves approached the plateau, indicating that the sequencing depth was sufficient (Fig. 1). The raw data of 16S rRNA gene sequence have been deposited in the GenBank data with the accession number: PRJNA890553.

The highest number of reads was found in the AMH1 sample (35,524), followed by the AFG1(33,573) sample, whereas the lowest number of reads was found in the AMH2 sample, with only 989 (Table 1).

**Table 1:** Summary of the bacterial diversity indices in coral samples: richness (ASVs and Chao1) and evenness (Shannon).

Coral Species	Status	Sample ID	Total Reads	ASVs	Chao1	Shannon
***Acropora formosa***	Grazed	AFG1	33,573	144	215.87	3.64
		AFG2	26,125	133	156.80	3.93
***Acropora millepora***		AMG1	31,463	152	236.64	3.92
		AMG2	28,587	175	234.37	3.92
		AMG3	23,482	152	209.19	3.83

Mean (standard deviation) for grazed coral samples				115.20(15.52)	209.77(31.51)	3.85(0.12)

***Acropora formosa***	Healthy	AFH1	4083	86	93.00	3.84
		AFH2	2986	121	127.65	4.16
		AFH3	31,891	206	316.56	4.28
***Acropora millepora***		AMH1	35,524	115	175.71	2.64
		AMH2	989	50	50.50	3.39

Mean (standard deviation) for healthy coral samples				115.00(56.67)	152.68(102.59)	3.66(0.67)

The number of ASVs ranged from 133 to 175 in the grazed samples and from 50 to 206 in the healthy samples. The mean bacterial diversities estimated by Shannon indices in the grazed and the healthy groups were 3.85 (SD 0.12) and 3.66 (SD 0.67), respectively. Calculations of alpha diversity revealed that the sample AFH3 had higher species richness (Observed: 206 and Chao1: 316.56) estimates than other samples, while those for AMH2 were the lowest (Observed: 50; Chao1: 50.50). However, there was no statistically significant difference in species richness between the grazed and healthy groups (*p* > 0.05). Similarly, the Shannon diversity index showed no significant difference between these comparisons (*p* > 0.05).

**Fig. 2:**
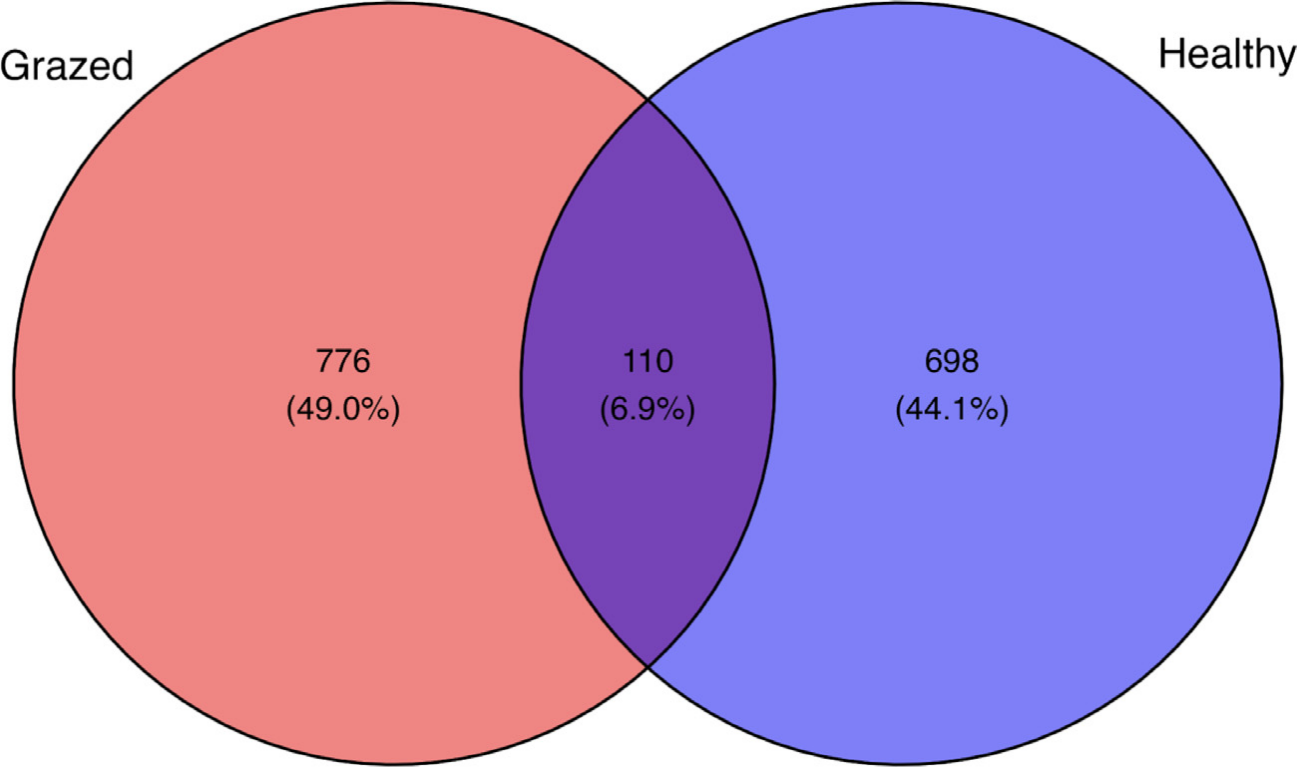
The venn diagramph showing the relationships between bacterial communities in the grazed and healthy groups.

The sequences were assigned amplicon sequence variants (ASVs) relying on the SILVA 16S rRNA gene database with a 97% cut-off similarity, generating a total of 1577 ASVs. Among these, the grazed samples contributed 776 ASVs, while the healthy samples generated 698 ASVs. We also discovered 110 ASVs that were shared by both the grazed and healthy samples (Fig. 2).

**Fig. 3:**
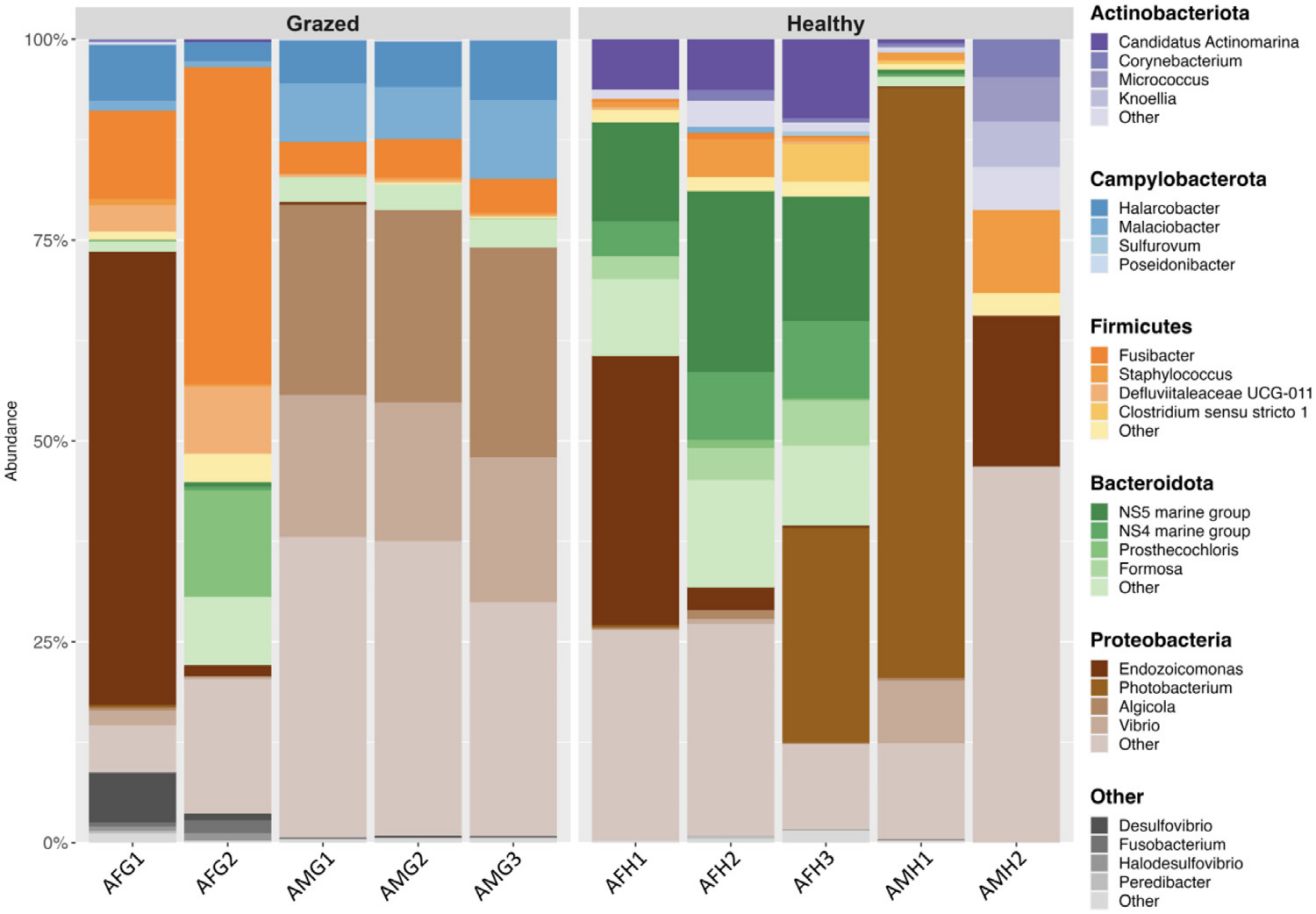
Taxonomic classification and top 5 bacterial taxa in relative abundance according to coral status.

The 1577 bacterial ASVs were then taxonomically classified into 19 phyla, 34 classes, 98 orders, 216 families, and 364 bacterial genera. Overall, the surveyed bacterial communities residing in the corals were dominated by the phyla *Proteobacteria, Bacteroidota, Firmicutes,* and *Actinobacteria*. As shown in Fig. 3, the top 5 bacterial phyla were displayed in the stacked bar plot. Accordingly, *Proteobacteria* were the most abundant in most samples (18.07–84.04%), followed by *Firmicutes* (1.70–50.58%), *Bacteroidota* (1.01–43.51%), and *Actinobacteria* (1.27–20.32%). *Firmicutes* were more abundant in the sample AFG2 (50.58%), while they were minor taxa in other samples, accounting for less than 17% of relative abundance. Besides, other phyla, including *Campylobacterota* (0.40-17.86%), *Desulfobacterota* (0.61-7.18%), *Fusobacteiota* (0.03-1.68%), and *Bdellovibrionota* (0.1-1.08%), were shown in lower abundances.

At the genus level, *Endozoicomonas, Photobacterium, Algicola*, and *Vibrio* were the ubiquitous genera in the phylum *Proteobacteria* and in most of the samples. The phylum *Actinobacteria* was dominated by *Candidatus Actinomarina,* whilst the phylum *Campylobacterota* was dominated by *Halarcobacter* (Fig. 3).

**Fig. 4:**
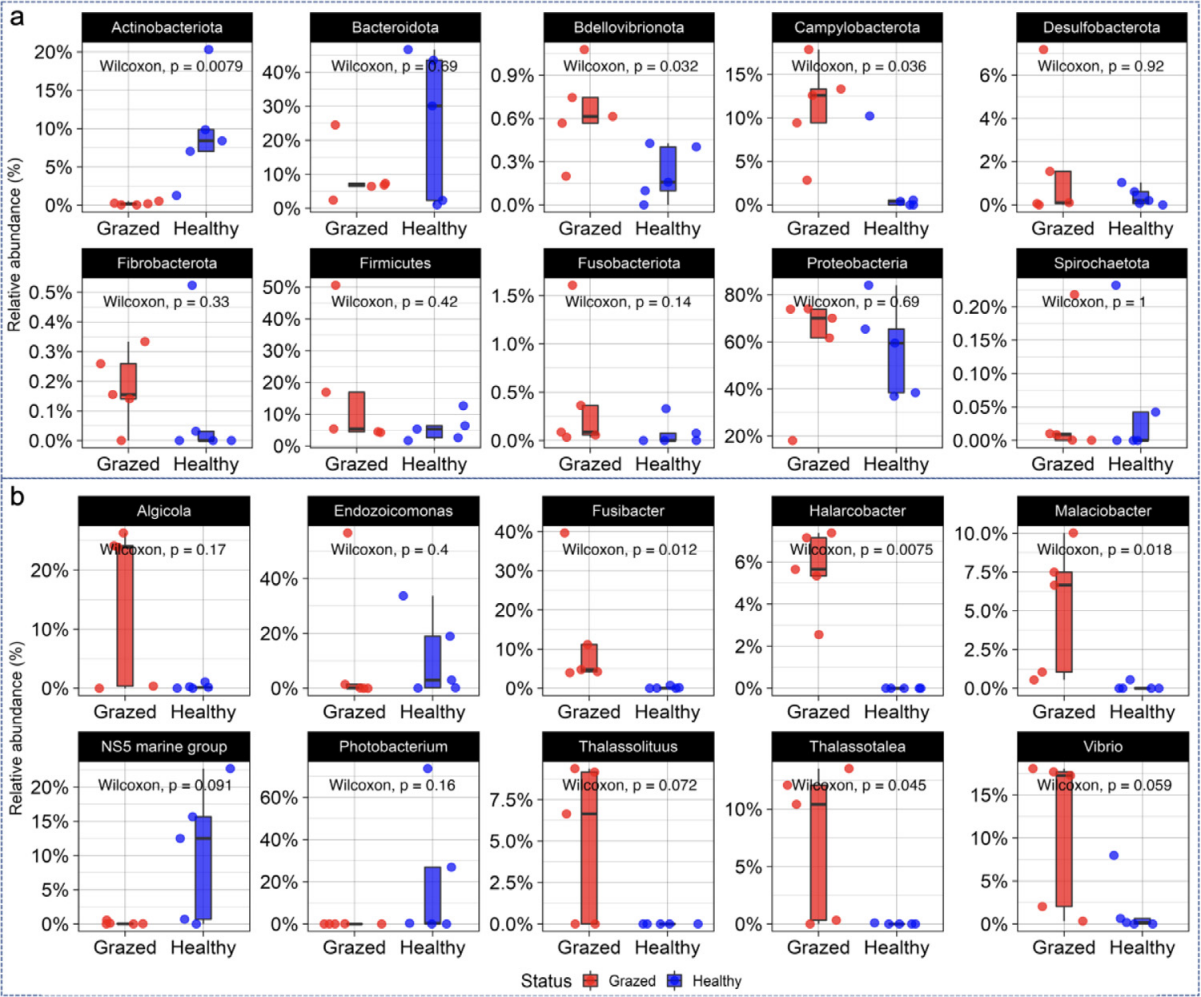
Statistical analysis (Wilcoxon rank-sum test) of the top 10 phyla (a) and genera (b) in two different coral sample groups (grazed and healthy).

A statistical analysis (Wilcoxon rank-sum test) of the top 10 genera revealed significant differences in the mean relative abundance of bacterial genera between the grazed and healthy samples. Specifically, the mean relative abundance of Fusibacter was higher (*p* = 0.012) in the grazed group (11.46 ± SD 13.7%) compared to that in the healthy group (0.24 ± SD 0.28%). Likewise, the genera *Halarcobacter, Malaciobacter,* and *Thalassotalea* in the grazed group had higher mean relative abundances than those of the healthy group (*p* < 0.05, Fig. 4). However, six genera, including *Algicola, Endozoicomonas, NS5 marine group, Photobacterium, Thalassolituus, and Vibrio,* had no significant differences in the mean relative abundances between the healthy and grazed groups (*p* > 0.05, Fig. 4).

**Fig. 5:**
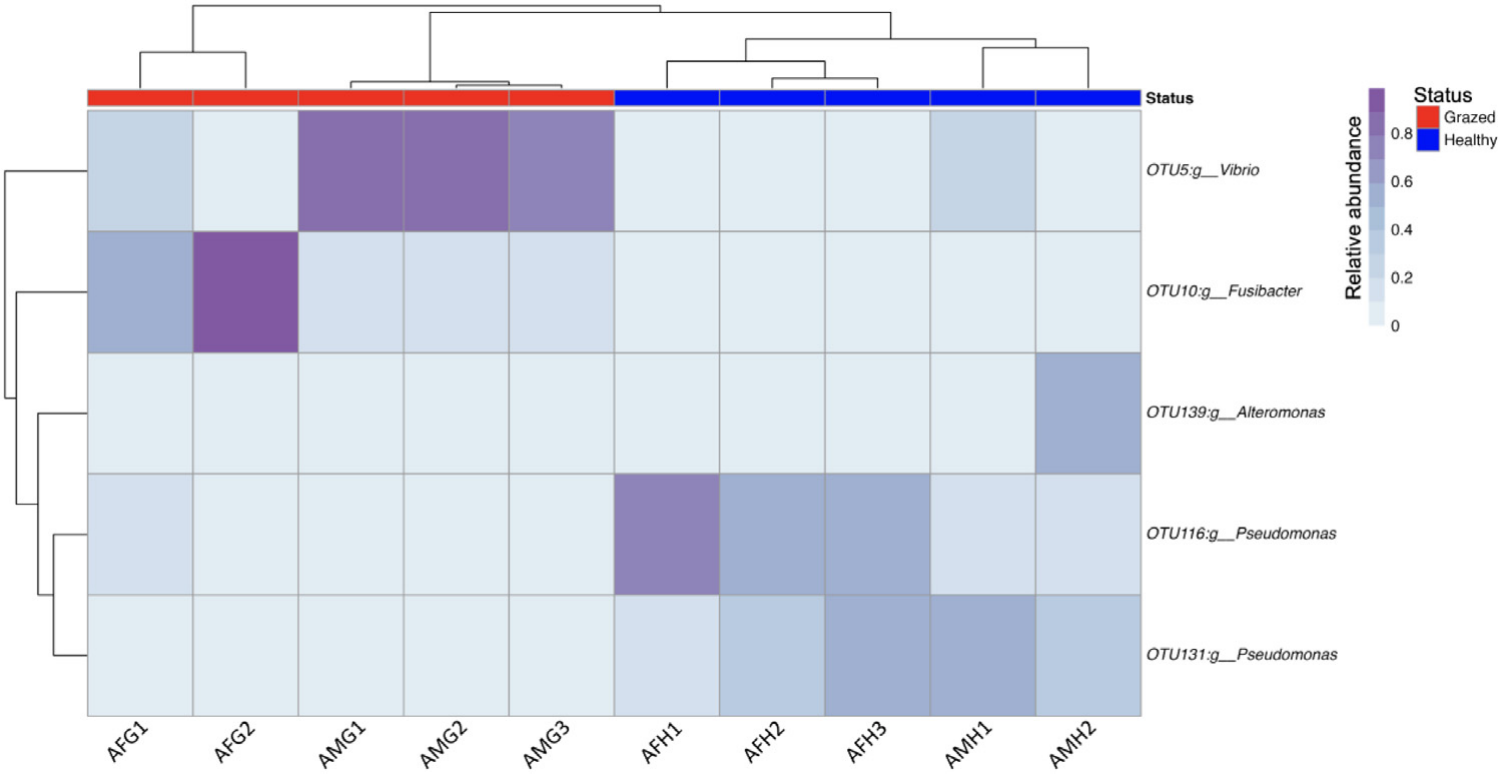
Heatmap of the core microbiome abundance across 10 samples.

In current study, an ASV was considered as a part of the core microbiome if it was present in at least 50% of samples. Using heatmap plot (Fig. 5), we identified a total of 5 ASVs considered as the core microbiome across all samples. These ASVs were classified at the genus level, including four genera, *Vibrio, Fusibacter, Altermonas,* and *Pseudomonas.*

As shown in Fig. 5, the genera *Vibrio* and *Fusibacter* dominated the core microbiome of the grazed samples, whereas, *Pseudomonas* was predominant in the healthy samples.

It is the first dataset on the bacterial communities in the grazed and healthy *Acropora* species, which was collected from Vietnam's Phu Quoc Islands and analyzed by 16S rRNA gene sequencing technology.

## 3 Experimental Design, Materials and Methods

### 3.1 Sample Collection

Colony fragments of *Acropora formosa (*3 for grazed, 2 for healthy*), Acropora millepora* (2 for grazed, 3 for healthy), widespread ubiquist specieses present in the global ocean were collected between 3 m and 5 m depth by scuba diving in shallow coral reefs of Hon Xuong Island of Phu Quoc Islands, Vietnam (9°55′20.6"N 104°01′16.4"E). Coral mucus was collected separately from grazed and healthy coral colonies. The fragment samples were taken out of the water with 3 minutes of air exposure. The mucus secretion that was triggered by this desiccation stress consisted of long gel-like threads dripping from the coral surface. The first 30 seconds of mucus production was discarded to prevent contamination and dilution by seawater. Then, the mucus samples were collected using sterile syringes and transferred from syringes to sterile cryotubes, where they were immediately fixed with 30% glycerol solution in a ratio of 1:1, then stored at –20°C until further use [1].

### 3.2 DNA Extraction, Library Preparation and Sequencing

DNA extractions were processed using the Easy-DNA™ gDNA Purification Kit (Invitrogen, Thermo Fisher Scientific, USA) following the manufacturer's instructions, and 500 µl bacterial DNA was extracted from different coral mucus samples. Amplification of the partial 16S rRNA gene with the purified template DNA was established by using the universal bacterial primer set 343F (S-D-Bact-0343- a-S-15, 5′-ACGGRAGGCAGCAG-3′) and 802 R (5′- TACCAGGGTATCTAATCCT-3′) [2], 2X PCR Taq Master Mix (Thermo Fisher Scientific, Waltham, MA, USA). These primers were attached with specific 6-bp barcode sequences at the 5′ end and then used for DNA amplification to produce 16S rRNA amplicons. A ∼460-bp fragment belonging to the V3–V4 region of the 16S rRNA gene was amplified. PCR amplifications were performed in an Eppendorf 6331 Nexus Gradient MasterCycler Thermal Cycler (Hampton, New Hampshire, USA) as follows: 30 cycles at 94°C for 5 min, 94°C for 30 s, 65°C for 30 s, 72°C for 1 min, and a final extension at 72°C for 2 min. All amplicons were checked for size and quality by agarose gel electrophoresis before using the Miseq Illumina platform to perform sequencing of the 16S rRNA gene. The purified PCR product was used to prepare the DNA library, following the DNA library preparation kit protocol.

### 3.3 Data Cleaning and Analyses

The protocol for processing raw sequence data reads included filtering and trimming low-quality sequences, denoizing, inferring sequence variants, constructing an ASVs table, and assigning taxonomy, as described by Callahan in 2016 [3]. Data analysis was performed using the DADA2 pipeline (version 1.8) – R Studio (version 4.2.1) with some modification to optimize accession of the dataset. Chimera filtering was performed by the “removeBimeraDenovo” function of the “dada2” package, while taxonomy was assigned using the Silva taxonomic training data formatted for DADA2 (SILVA ribosomal RNA gene database project version 138.1) [4].

Alpha diversity metrics were calculated to compare the microbial diversity of the samples, which included observed ASVs, the Chao1 richness estimator, and the Shannon–Weaver index, using the Vegan package in R [5]. As the majority of datasets did not follow an assumption of normality distribution, the Wilcoxon rank-sum test was used to compare the difference in relative abundance of bacterial taxa (phyla and genera) between two grazed and healthy groups. A *p*-value of <0.05 was considered statistically significant.

The core microbiomes of coral genus were identified using the “microbiome” package and were illustrated with a Venn diagram. The core ASVs in coral microbiomes have been defined using different percentage cut-offs ranging from 30% to 100% [6]. In the current study, the presence of ASVs in at least 50% of samples was chosen as a conservative representation of the core microbiome.

## Ethics Statements

This dataset has no involvement to human or animal ethics.

## CRediT Author Statement

**Duong Huy Nguyen:** Methodology, Software, Formal analysis, Data curation, Writing – original draft, Writing – review & editing; **Nhat Huy Chu:** Project administration, Funding acquisition, Writing – review & editing; **Yvan Bettarel:** Project administration, Resources, Writing – review & editing; **Jean-Christophe Auguet:** Sampling, Methodology, Writing – review & editing; **Thierry Bouvier:** Sampling, Investigation, Writing – review & editing; **Ha Hoang Chu:** Supervision, Resources, Writing – review & editing; **Van Ngoc Bui:** Conceptualization, Supervision, Project administration, Funding acquisition, Resources, Writing – review & editing.

## Declaration of Competing Interest

The authors declare that they have no known competing financial interests or personal relationships that could have appeared to influence the work reported in this article.

## Data Availability

Raw metagenomic dataset (Original data) (Mendeley Data).
